# Association Between Physical Activity and Fitness in Patients with Heart Failure and Type 2 Diabetes Mellitus: Influence of a Telemedicine Program

**DOI:** 10.3390/healthcare13243250

**Published:** 2025-12-11

**Authors:** Mara Paneroni, Palmira Bernocchi, Beatrice Salvi, Carla Simonelli, Gloria Fiorini Aloisi, Luigina Viscardi, Salvatore D’Isa, Simonetta Scalvini

**Affiliations:** 1 Cardio-Respiratory Rehabilitation Unit of the Institute of Lumezzane, Institutes Clinici Scientifici Maugeri IRCCS, 25065 Lumezzane, Brescia, Italy; beatrice.salvi@icsmaugeri.it (B.S.); carla.simonelli@icsmaugeri.it (C.S.); 2 Continuity of Care Unit of Institute of Lumezzane, Istituti Clinici Scientifici Maugeri IRCCS, 25065 Lumezzane, Brescia, Italy; palmira.bernocchi@icsmaugeri.it (P.B.); gloriaafiorini@gmail.com (G.F.A.); simonetta.scalvini@icsmaugeri.it (S.S.); 3 Department of Cardiac Rehabilitation, Bolognini Hospital, Azienda Socio-Sanitaria Territoriale Bergamo Est, 24068 Seriate, Bergamo, Italy; luigina.viscardi@asst-bergamoest.it; 4 Cardiology Unit, Cardiovascular Department, ASST Papa Giovanni XXIII, 24127 Bergamo, Bergamo, Italy; sdisa@asst-pg23.it

**Keywords:** heart failure, diabetes mellitus type 2, tele-health, telemedicine, teleassistance, telemonitoring, telerehabilitation, motivational feedback, physical activity, 6-min walking test

## Abstract

**Highlights:**

- Physical fitness (PF) and physical activity (PA) are closely related in individuals with heart failure and type 2 diabetes mellitus, but our telemedicine program only moderately impacted PF. Specific baseline factors influenced outcomes after six months of intervention.
- Younger patients with higher baseline PF, HDL cholesterol levels, and PA showed better PA outcomes at the end of the program. Meanwhile, those with lower BMI, younger age, and better baseline PF demonstrated greater improvements in PF.

**What are the main findings?**

- After our telemedicine program, the 6-Minute Walking Test (6MWT) improved, while PA levels remained unchanged.
- The association between PA and PF increased from moderate at baseline to strong at the end of the program.
- PF, age, HDL cholesterol, and baseline PA were key predictors of PA, while BMI, age, and baseline 6MWT influenced PF outcomes.

**What are the implications of the main findings?**

- PA and PF are distinct parameters that should be measured and monitored independently.
- Telemedicine programs should include targeted interventions to improve both PF and PA. Stratifying patients based on specific baseline characteristics may support selecting the most appropriate program, enhancing personalization and effectiveness.

**Abstract:**

**Background.** Few studies have evaluated physical fitness (PF) and physical activity (PA) in individuals with heart failure and type 2 diabetes mellitus and the possibility that some telemedicine programs (TMPs) may impact them. This post hoc subgroup analysis of an RCT study aimed to describe PF and PA in this population before and after a TMP. **Methods.** We evaluated (a) PF as distance, assessed via the 6-Minute Walking Test (6MWT), and PA as daily step count in this population before and after a TMP; (b) their relationship; and (c) the patient characteristics that influence PF and PA. **Results.** Fifty-eight patients (aged 71.31 ± 7.92 years old, 84% male, BMI 28.01 ± 4.70 Kg/m^2^, ejection fraction 48.64 ± 10.64%) were enrolled between August 2022 and September 2024. All patients received a six-month TMP (nurse teleassistance, telemonitoring, a dedicated app, and PA evaluation using a tracker bracelet and step count goals). The 6MWT improved (from 418 ± 113 to 439 ± 120 m, *p* < 0.001), while PA remained unchanged after the TMP (from 7181 ± 4149 to 7229 ± 4947 steps/day, *p* = 0.92). The PA and PF association ranged from moderate at baseline (rho = 0.4958, *p* < 0.001) to strong at the end of the study (rho = 0.6807, *p* < 0.001). The regression model shows that following the TMP, PA(y) was associated with baseline 6MWT [β= 8.5, 95%IC −0.31; 17.24], age (β = −144.0, 95%IC −262.14; −25.90)), baseline daily steps (β = 0.5967, 95%IC 0.37; 0.82), and HDL cholesterol (β = 119.7, 95%IC 39.07; 200.31) (R^2^= 0.6580, F(4.53) = 25.49, *p* < 0.001), while PF(y) was associated with BMI (β= −2.04, 95%IC −4.30; 0.22)), age (β= 0.90, 95%IC −4.4; 1.32), and baseline 6MWT (β = 0.90, 95%IC 0.79; 1.00) (R^2^ = 0.9007, F(3.54) = 163.29, *p* < 0.001). **Conclusions**. Our TMP led to a statistically significant but clinically modest improvement in PF but did not impact PA despite there being variability among patients. PA and PF appear to be interdependent. PF, age, HDL cholesterol, and baseline PA were key predictors of PA, while BMI, age, and baseline 6MWT impacted PF at the end of the TMP.

## 1. Introduction

In recent years, the prevalence of chronic multi-morbidities has dramatically increased due to the progressive aging of the population. Heart failure (HF) is a major public health problem affecting 1–2% of adults, and prevalence increases with age, reaching over 10% in individuals aged 70 years or older [1].

A particularly relevant comorbidity among HF patients is type 2 diabetes mellitus (T2DM) [2].

In clinical studies, the prevalence of T2DM is approximately 30% in patients with either reduced or preserved ejection fraction HF, rising to 45% in hospitalized patients [3,4]. T2DM in HF patients is associated with poorer functional status, worsening renal and endothelial function, impaired gas exchange, a worse prognosis, and a lower quality of life compared to HF patients without diabetes [5,6,7,8].

The management of these chronic conditions requires a multifaceted approach that encompasses both pharmacological therapies and comprehensive strategies to enhance self-management and promote behavioral modifications. These modifications include dietary regulation, restriction of sodium intake, and systematic monitoring of body weight and blood glucose levels [9].

Among these lifestyle modifications, increasing physical activity (PA) and physical fitness (PF) are the most important steps. In this context, PF refers to the ability to tolerate an activity or exercise, while PA refers to the total amount of movement in daily life.

In patients with HF, daily PA was found to be positively related to peak VO_2_ and to independently predict mortality [10]. Additionally, diabetes mellitus may reduce daily physical activity in patients with HF [11].

A recent prospective study of patients with HF and reduced ejection fraction showed how a simple lifestyle walking intervention combining self-monitoring with an activity tracker and telephone counseling resulted in higher engagement in PA, but it did not improve patients’ PF [12]. Conversely, exercise training programs, typically included in cardiac rehabilitation and telerehabilitation, are capable of improving PF but do not automatically increase subjects’ PA levels unless they include counseling and behavioral coaching components [13,14]. In this context, a dedicated telemedicine program (TMP) that includes routine self-management support, electronic information systems for sharing patient data among engaged healthcare professionals, telemonitoring, and stimulation for healthcare professionals was able to promote continuity of care, reduce disability, and improve both PA and PF in elderly and high-risk multimorbid populations [15,16].

To our knowledge, there is a specific research gap regarding the relationship between PA and PF in people affected by both heart disease and T2DM and how TMPs may affect them. This is a particularly compelling population to study long-term management through telerehabilitation programs as these individuals share overlapping pathophysiological mechanisms and management challenges, and they have synergistic clinical risks that underscore the need for integrated, technology-assisted care models. In this context, evaluating the functional capacity of patients and responsiveness to home-based telemedicine programs is of paramount importance to identify the most effective intervention and the most suitable setting/program and technological approach.

This study aimed to describe PF, assessed via the 6-Minute Walking Test (6MWT), and PA, measured as daily step count, in a cohort of clinically stable adults with both HF and T2DM before and after a TMP. Secondary objectives were to identify novel findings regarding (a) the relationship between PA and PF at baseline and after the TMP, as well as the association between changes in PA and PF over time, and (b) the baseline anthropometric and clinical characteristics that influence PF and PA following a TMP.

## 2. Materials and Methods

This is a post hoc analysis of a randomized controlled trial (RCT) conducted in three Italian hospitals (Istituti Clinici Scientifici Maugeri IRCCS, Institute of Lumezzane, Brescia, Italy; Bolognini Hospital, Seriate, Bergamo; and Papa Giovanni XXIII Hospital, Bergamo) [17].

The study was approved by the local institutional review board and the Ethical Committee at each study site. It was registered on 30 November 2022 at http://www.clinicaltrials.gov (NCT05633784) and was conducted in accordance with the recommendations for interventional trials, the SPIRIT and CONSORT guidelines, the principles of the Declaration of Helsinki, and good clinical practice.

We analyzed subjects allocated to the study group (intervention arm) of this RCT (TMP, see below) who had undergone both PA and PF assessments. No control group data were available; we aimed to focus on these peculiar aspects. Patients were enrolled during a follow-up visit for HF and were included if they met the following criteria: a diagnosis of HF with a reduced or preserved ejection fraction and T2DM, age ≥ 18 years, stable condition without hospitalization due to decompensated HF in the past three months, and the ability to walk without aids. Patients were excluded if they had (a) a life expectancy of less than six months, as defined by the cardiologist in light of the patient’s clinical condition, and (b) the impossibility of using mobile technology.

### 2.1. Telemedicine Program

In addition to standard care, including visits to the general practitioner and hospital control upon request, patients were followed through a home-based TMP for six months. The TMP was performed as follows: upon enrollment, the patients received a tracker bracelet, an electrocardiogram device, and a smartphone with pre-installed apps to receive information from wearable devices.

Patients were encouraged to engage in regular physical activity under the guidance of a dedicated healthcare professional (HP) (nurse and/or physiotherapist) through scheduled video calls held three times a week. These sessions emphasized the importance of lifestyle changes and consistent exercise. Patients were instructed to wear their Fitbit devices (Fitbit Inspire 2) (Fitbit Inc., Google LLC, Mountain View, CA, USA) at all times to continuously monitor their daily step count.

The HP tracked each patient’s daily step count via the Health Platform web portal (CompuGroup Medical SE, CGM, Milan, Italy). In cases of non-adherence or any other issues, the HP scheduled additional video calls to address the problems and provide support.

Each patient was given a personalized step goal. Based on their baseline activity level, patients were encouraged to increase their daily step count by 10% every two weeks according to our daily/step progression protocol tested previously [18]. The HP monitored progress remotely. If a patient failed to meet the step target for two consecutive working days, the HP contacted them via additional video or phone calls to provide guidance and encouragement to promote a healthy lifestyle. Considering the need to modify behavior, we arbitrarily defined non-adherence as failure to perform the prescribed activity for two consecutive working days.

The following indicators were used to assess adherence to PA and PF during the study period: the number of teleconsultations per patient; whether the Fitbit was worn for at least 12 h per day (yes/no); and adherence to step goals.

In addition to the aforementioned activities, the TMP was characterized by the following:

1. Support was provided by a nursing case manager through a structured teleassistance program (phone or video calls at least once a week) and telemonitoring of patients’ vital signs (e.g., single electrocardiographic trace).
2. Cardiology and diabetes teleconsultations were available at the beginning and throughout the program in case any clinical problems arose.
3. A dedicated app was available to record and monitor drug treatments and clinical parameters every day (see below).

The HP was able to remotely manage enrolled patients’ treatment paths by prescribing therapies and tasks, viewing the progress of clinical parameters collected and entered by patients (e.g., vital signs and symptoms), interacting with patients by exchanging text messages and multimedia files, activating videoconferences, and delivering questionnaires. A detailed description of the TMP can be found in [17].

### 2.2. Health Information Technology Components

The protocol utilized two main health information technology components:

- The Health Platform web portal and app (CompuGroup Medical SE, CGM, Milan, Italy) (Platform A): The Health Platform is a software consisting of a web portal used by healthcare personnel to manage patients. It acquires vital parameters through a three-lead Hi-ECG device (CompuGroup Medical SE, CGM, Milan, Italy), which transmits electrocardiogram traces to a smartphone via Bluetooth. The collected data are saved in the smartphone’s internal app (CompuGroup Medical SE, CGM, Milan, Italy) database and associated with the user. This smartphone app is a gateway to recognize the associated ECG and sends the data to the server via the Health Platform. PA data is recorded using a tracker bracelet (Fitbit Inspire 2) and is automatically transported to the Fitbit app and server, and it is retrieved daily from the Health Platform server. The HP could access a specific section of Platform A using personal credentials to view patients’ electrocardiographic traces and Fitbit server data.
- The TreC Cardio web portal and app (Fondazione Bruno Kessler, Trento, Italy) (Platform B): The “TreC Cardio” platform includes a web dashboard for healthcare personnel to manage patients. The app is available on Android/iOS (Fondazione Bruno Kessler, Trento, Italy) and allows patients to collect clinical data and communicate with doctors and nurses. Patients can use the “TreC Cardio” app (Fondazione Bruno Kessler, Trento, Italy) to view their medication history and upcoming doses, confirm whether they have taken their daily therapy, record self-detected clinical parameters and symptoms, and receive reminders for healthcare actions (e.g., measuring blood pressure). A dedicated chat allows users to send images/PDFs and make video calls with healthcare personnel.

### 2.3. Measures

The following information relevant to this study was evaluated:

At baseline (T0), demographic, clinical, and quality of life variables were collected. The following variables were evaluated before and after six months (T0–T6) of the TMP:

- PF, as measured using the 6MWT [19], was performed according to the ERS/ATS statements. The predicted value of the test was calculated using Enright and Sherill’s formula [20], which included data regarding patients’ sex, weight, and height. The test was performed at the beginning and end of the study period.
- PA, as measured by daily step count, was objectively measured using a Fitbit Inspire 2 activity tracker. Participants were instructed to wear the tracker for at least four consecutive days, including during sleep, at the beginning and end of the TMP. The HP remotely monitored adherence to device use and verified that the device was worn for the required duration. If data recording was interrupted for more than two consecutive hours, patients were advised to wear the device for an extended period to achieve at least 3 days of uninterrupted monitoring within ten days of the start of monitoring. Missing data were excluded from the analysis and not considered when calculating outcomes; we only analyzed continuous monitoring traces

### 2.4. Statistical Analysis

We analyzed data using STATA 12.4 software (Stata Corp LLC, College Station, TX, USA) and tested data normality using the Shapiro–Wilk test. Variables that were normally distributed are described by the mean ± standard deviation, while non-normally distributed variables are described by the median and 1st and 3rd quartiles. Categorical variables are described by absolute and relative frequencies (%). We evaluated pre-to-post differences within groups using the paired *t*-test for normally distributed variables and the Wilcoxon test for non-parametric variables. Differences between groups were evaluated using an unpaired *t*-test for normally distributed variables and a Mann–Whitney U-test for non-parametric ones. The effect size was calculated for comparisons between means using Cohen’s d and for comparisons between medians using rank biserial correlation (rrb), but only if the results were significant. Effect size was defined as small (d = 0.1), medium (d = 0.3–0.6), or large (d ≥ 0.7)] for Cohen’s d and small (rrb = 0.1–0.3), medium (rrb = 0.3–0.5), or large (rrb ≥ 0.7) for rank biserial correlation.

Spearman ’s correlation (rho) was used to assess the relationship between PA and PF. Values of ρ < 0.30 were interpreted as indicating a weak association between variables, 0.30 ≤ |ρ| < 0.50 as moderate, and |ρ| ≥ 0.50 as strong.

At baseline, patients were categorized into groups based on two thresholds: 5000 steps per day for physical activity (PA), which was used to categorize high or low PA [21], and 70% of the predicted value of the 6MWT, which was used to categorize high or low PF [22]. Considering changes in PF and PA, patients were assigned to two groups based on improvements or worsening of their daily step count over the study period. The cut-off value was 600 steps, which is the Minimal Clinically Important Difference (MCID) evaluated in other chronic populations [23,24]. The MCID for the 6MWT was set at 32 m [25].

A multiple linear regression model was employed to identify independent associations between PA and PF (dependent variables: daily steps and meters at 6MWT, respectively) at the end of the TMP and baseline clinical and anthropometric variables [independent variables: sex, age (years), BMI (kg/m^2^), ejection fraction (%), baseline 6MWT, baseline daily step count, SF-12 PCS, SF-12 MCS, PASE, and blood analysis parameters (glycemia, glycated hemoglobin, NT-proBNP, creatinine, total cholesterol, LDL cholesterol, and HDL cholesterol)]. Standardized beta coefficients (β) were reported to evaluate the relative contribution of each predictor variable, allowing for comparison across variables with different units of measurement. The regression model assumptions of linearity, independence, homoscedasticity, and normality of residuals were assessed using graphical analysis and the Shapiro–Wilk test. Multicollinearity among the independent variables was evaluated using variance inflation factors (VIFs), with values greater than five indicating potential collinearity. All assumptions were satisfied for both regression models. There was no missing data identified for the main parameters assessed in this study, and no patients were excluded as outliers. *p* < 0.05 was considered significant.

## 3. Results

We analyzed the data of 58 patients enrolled between August 2022 and September 2024 (n = 38, Lumezzane (BS); n = 13, Seriate (BG); n = 7, Bergamo).

Table 1 shows the demographic and clinical characteristics of the study population. The sample primarily consists of older adults, with a higher prevalence of male participants (84%). Based on BMI, most individuals are considered overweight. Polypharmacy is common among participants, with a significant proportion requiring three or more medications to manage their conditions. Most individuals exhibit moderate (NYHA class II) heart failure, while only a small subset of patients present with either mild or severe symptoms. Blood biomarkers indicate the presence of metabolic and cardiovascular risk factors, including hyperglycemia, and elevated circulating markers of heart failure reflect a significant cardiac burden. Additionally, indicators of renal function suggest mild to moderate impairment, and lipid profile assessments reveal variability among participants, with a general trend toward dyslipidemia. Quality of life evaluations highlight a heterogeneous impact of the disease. Overall, there was a mild reduction in the PF condition and PA profile despite high variability among the samples.

During the six-month study period, the number of teleconsultations per patient was 21 ± 7.5, the Fitbit was worn for at least 12 h per day in 100% of cases, and the rate of adherence to step goals was 88%.

**Figure 1:**
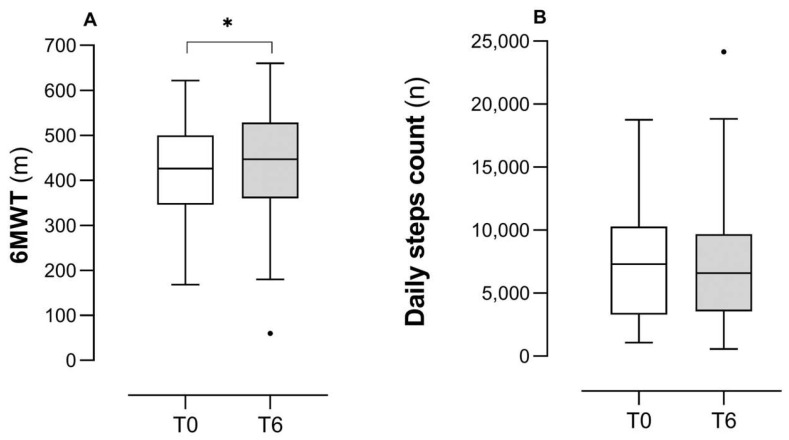
Changes in PF and PA following a TMP. Legend: Panel (**A**): 6MWT = 6-Minute Walking Test; m = meters; Cohen’s d = 0.49. Panel (**B**): n = number of daily steps. * is for *p* < 0.001, the dot represents the outlier data.

Figure 1 describes PF assessed via the 6MWD (panel A) and PA (panel B) measured with daily step count before and after a TMP intervention in the studied group. The increase in 6MWT was statistically significant (from 418 ± 113 to 439 ± 120 m, *p* < 0.001) with a mean increase of 21 m (SD of differences = 43) and a medium effect size (Cohen’s d = 0.49). Post-estimation of power indicated that the sample size of 58 was sufficient (power ≈ 0.95). Conversely, PA did not change after the TMP intervention (from 7295 (3368–10,278) to 6579 (3615–9577) steps, *p* = 0.85).

**Figure 2:**
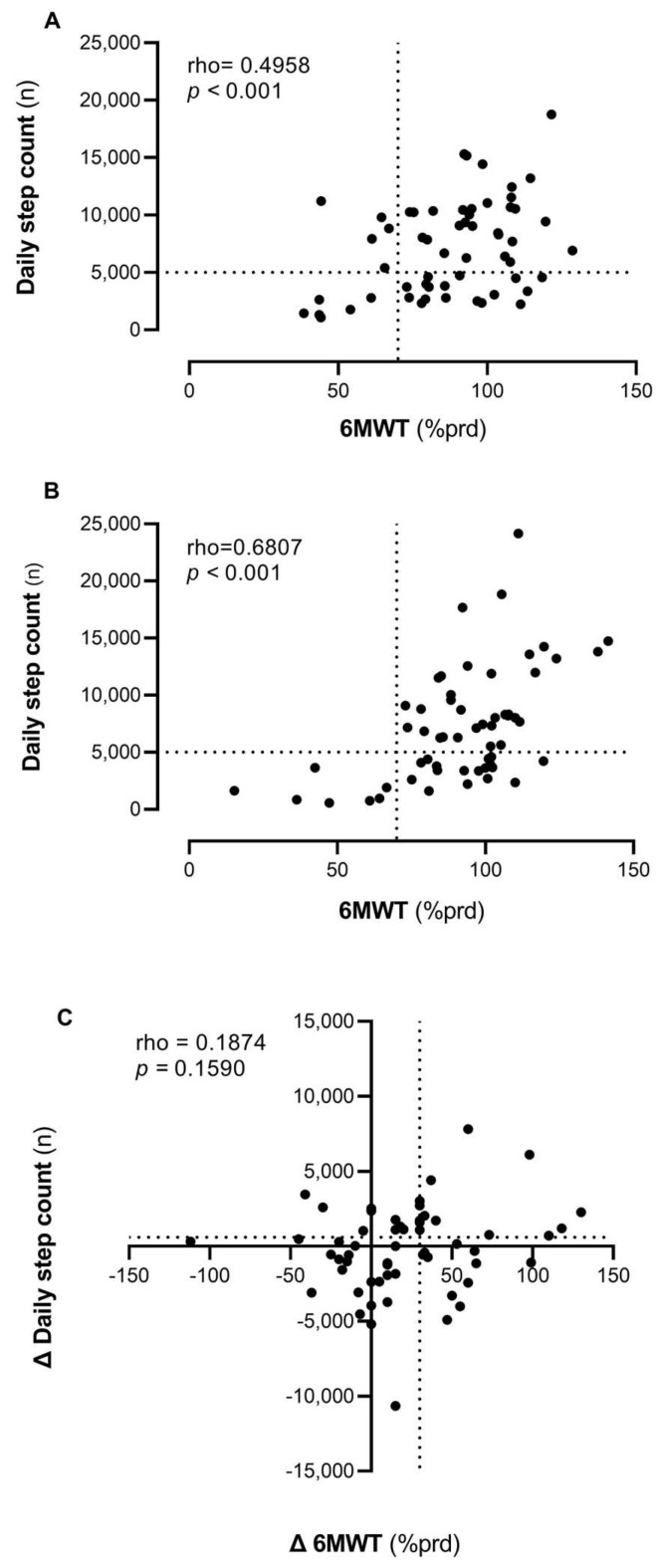
Correlations between PA (daily step count) and PF (6MWT) at baseline (Panel (**A**)) and after TMP (Panel (**B)**) and the association between their changes over time (Panel (**C**)). Legend: 6MWT = 6-Minute Walking Test; % prd = percentage of predicted value.

Figure 2 (panels A and B) describes the correlation between PA and PF at baseline and at the end of rehabilitation. The correlation ranged from moderate (rho 0.4958, *p* < 0.01) to strong (rho 0.6807, *p* < 0.001). However, there is significant variability in daily steps among patients with similar PF levels. Initially (panel A), half of the patients (52%) are in the quadrant characterized by both high PF and high PA levels. A total of 29% of patients have high PF but engage in low PA, walking fewer than 5000 steps per day. Among the remaining patients, 10% have both poor PF and low PA, while 9% have reduced PF but manage to walk more than 5000 steps daily. By the end of the program, the correlation between daily steps and meters walked in the 6MWT remains significant and becomes stronger.

Figure 2 (panel C) shows how changes in PF and PA are related to the TMP intervention. The correlation between these two modifications is low and not statistically significant (rho = 0.1874, *p* = 0.16). Some participants (n = 43, 74%) improved in the 6MWT, with a subset (n = 20, 34.48%) exceeding the MCID threshold of 32 m [24]. Regarding physical activity, approximately half of the individuals (n = 30, 52%) exhibited improvement, with some (n = 24, 41.38%) surpassing the MCID of 600 steps [22,23].

Seventeen percent (n = 10) of participants improved in both parameters, exceeding the respective MCID thresholds, which accounts for 17.2% (n = 10) of the sample. Lastly, 46.6% (n = 27) of patients showed improvement in only one of the two parameters, and 36.2% (n = 21) did not improve in either parameter.

Table 2 compares patients who increased their PA (daily step count) beyond the MCID threshold (600 steps) and those who did not. Patients who demonstrated improvement were younger and exhibited a higher physical component score (PCS) on the Short-Form 12 (SF-12) questionnaire. Blood analyses revealed statistically significant differences in glycated hemoglobin, renal clearance, and HDL cholesterol levels between groups, with patients who did not improve displaying poorer metabolic and renal profiles.

Considering the baseline variables described in Table 1, we performed a linear regression model (Table 3) to describe the measures that were significantly associated with PA (R^2^ = 0.6580, *p* < 0.01) and PF (R^2^ = 0.9007, *p* < 0.001) following the TMP intervention. Regression assumptions were satisfied for both models.

## 4. Discussion

Patients with heart failure and type 2 diabetes who participated in the TMP intervention showed a modest, though statistically significant, improvement in physical function. No relationship was found between changes in PA and PF, and no significant change was observed in physical activity. At six months, higher PA levels were associated with greater baseline step counts, better baseline 6MWT performance, younger age, and higher HDL levels. Higher PF was associated with lower BMI, younger age, and longer baseline 6MWT distance.

Few studies have examined similar patient populations. Dunbar et al. [26] evaluated a self-management intervention for patients with heart failure and type 2 diabetes, combining education, counseling, home visits, and telephone follow-ups. Compared with standard educational materials, the intervention led to higher physical activity levels, greater six-minute walk distances, and improved heart failure-related quality of life and physical activity.

While the findings related to PF in the treated group are consistent with our results, those concerning PA differ. Notably, in the study by Dunbar et al. [26], PA was assessed solely through self-report questionnaires, which may not accurately capture actual changes in physical activity behavior, such as step count. This differs from our study, which used objective device data. Furthermore, the patients enrolled in the Dunbar study [26] had recently been hospitalized for HF, whereas the participants in our study had stable clinical conditions. This important distinction may have significantly influenced the outcomes.

Evidence on TMPs in this patient population is still limited. More generally, there is no consensus on the most effective intervention model for managing chronic disease. Digital health interventions appear promising in this context: a recent systematic review of 14 randomized controlled trials (n = 1497) reported that such interventions significantly improved physical activity after cardiac rehabilitation (SMD = 0.35; 95% CI 0.02–0.70; *p* = 0.04) [27].

It is notable that digital health interventions for cardiovascular disease secondary prevention are typically very heterogeneous—including building blocks such as websites, text messages, wearable sensing devices, smartphone apps, and virtual reality programs—and little information is available on their underlying program theory, i.e., what intervention components are expected to lead to which changes [27].

Evidence from studies on chronic respiratory conditions has yielded mixed results, with variability in how different programs affect changes in both PA and PF [22].

Digital health interventions that aim to support behavioral change are often complex, combining several components to target multiple behaviors. Developers of these complex interventions should ‘articulate program theory’, describing all individual intervention components (‘active ingredients’) and their proposed mechanisms of action. They should also explain the rationale(s) for each intervention component and proposed mechanism of action, including the underpinning theories and/or empirical evidence [28]. A personalization algorithm (e.g., stratification by age, BMI, baseline physical function/physical activity, and HDL levels) could represent an effective operational strategy for tailoring the intervention program to this patient population’s specific characteristics.

These findings highlight the relevance of personalized telerehabilitation strategies that account for patient heterogeneity to maximize adherence and long-term benefits in chronic cardiac disease management. Our program integrated telemonitoring (TMP) with a step count goal, an approach previously shown to be beneficial in some settings [29]. However, only a small proportion of participants in our study increased their PA, indicating that step-based targets alone may be insufficient to induce meaningful behavioral change in this population. Incorporating cadence control as an additional behavioral target could be a more effective strategy because cadence directly relates to exercise intensity and supports engagement in moderate-to-vigorous activity [30]. It should be evaluated as an enhancement to step count monitoring. Such an approach could be effectively implemented through telemonitoring, enabling real-time assessment of symptoms, heart rate, and exercise intensity. Our data suggest that individuals with an adequate baseline level of physical functioning and a pre-existing habit of being physically active are more likely to improve their PA following the intervention. Additionally, a more favorable lipid profile was associated with greater increases in PA, potentially reflecting overall better control of cardiovascular risk factors.

However, even when physical capacity is preserved, some patients demonstrate markedly low levels of actual PF. This suggests that behavioral, psychological, or environmental factors may override physiological potential in determining real-world activity levels.

Nevertheless, regression analysis indicated that patients with better 6MWT performance at baseline, a lower body mass index, and a younger age were more likely to show improvements in PF.

### 4.1. Limitations

The main limitation is that the study is post hoc of an RCT. In the absence of a control group, the study is primarily descriptive. Additional limitations of this study include the following: (i) We did not formally assess facilitators or barriers to physical activity (e.g., potential seasonality effects, social context, etc.), even though the TMP addressed these aspects during the intervention. (ii) The sample size is relatively small, which may affect the generalizability of the findings. (iii) The conducted analyses are exploratory and have a post hoc design without a comparator and should be interpreted with caution. (iv) The 6MWT can only be seen as a surrogate for the cardiopulmonary exercise test (CPET), which is the gold standard for defining physical fitness. Thus, the 6 MWT can suffer from a ceiling or floor effect. In this context, future studies should investigate the relationship between PA and PF using the CPET as a reference, as different results may emerge [31]. Additionally, (v) due to the absence of robust cut-offs of normality for daily steps and 6MWT (percentage of predicted) in our population, we used those referred to other chronic populations (i.e., COPD), and (vi) we evaluated PA over a short monitoring window; different response patterns might have been observed with a longer monitoring period. This aspect should be considered, and data should be interpreted cautiously.

### 4.2. Clinical Implications

The findings of this study suggest that a TMP with step count goals may not be equally effective in improving overall PA and PF in patients affected by heart failure and type 2 diabetes. Future programs may benefit from a more personalized approach, integrating physiological targets (e.g., cadence, heart rate target, and perceived exertion target) alongside behavioral goals to stimulate improvements in physical functioning more effectively. Additionally, the identification and structured assessment of individual barriers and motivators could enhance the effectiveness of behavioral interventions.

## 5. Conclusions

In patients with heart failure and type 2 diabetes, physical activity and physical function are interrelated and influenced by distinct factors. Higher physical activity at six months was associated with greater baseline step counts, longer 6MWT distance, younger age, and higher HDL levels. Higher physical function was associated with lower BMI, younger age, and longer baseline 6MWT distance. These findings highlight the importance of tailoring rehabilitation interventions to individual patient profiles to optimize outcomes. Telemedicine programs should incorporate interventions that target improvements in both domains. Stratification according to the specific issue identified may enhance program selection and enable greater personalization.

## Figures and Tables

**Table 1:** Demographic and clinical characteristics of the study population.

Measures	
Patients, n	58
Sex, n (%)	
Male	49 (84%)
Female	9 (16%)
Age, years	71.31 ± 7.92
BMI, kg/$m^{2}$	28.01 ± 4.70
Number of drugs, n (%)	
≤2	15 (26%)
3	17 (29%)
4	16 (28%)
≥5	10 (17%)
NYHA classification, n (%)	
1	1 (2%)
2	35 (60%)
3	21 (36%)
4	1 (2%)
EF %	48.64 ± 10.64
EFr, n (%) EFc, n (%)	13 (22.41%) 45 (77.59%)
6MWT, meters	417.95 ± 112.89
6MWT, % of the predicted value	87.97 ± 21.73
Daily step count, n	7295 (3368–10,278)
**Questionnaires**, score	
MLHFQ	9.5 (2–17)
SF-12: PCS	42.60 ± 10.01
SF-12: MCS	54.80 ± 6.49
PASE	85.41 ± 46.13
DQOL Section 1	28.19 ± 7.83
DQOL Section 2	7 (25–30)
DQOL Section 3	6 (5–8)
**Blood chemistry tests**	
Glycemia, mg/dL	137.5 (111–146)
Glycated hemoglobin, mmol/mol (NCR < 42 mmol/mol)	50 (46–63)
NT-proBNP, pg/mL	850.5 (178–1001)
Renal clearance, mL/min	59.41 ± 23.36
Creatinine, mg/dL	1.22 (1.06–1.47)
Total cholesterol, mg/dL	122 (109–151)
LDL cholesterol, mg/dL	57.5 (48–83)
HDL cholesterol, mg/dL	42 (37–46)

Legend: Data are presented as mean ± SD or median (1st–3rd quartile) or number (%). BMI: Body Mass Index; NYHA: New York Heart Association Functional Classification; EF: Ejection Fraction; EFr: Ejection Fraction Reduced; MLHFQ: Minnesota Living with Heart Failure Questionnaire; SF-12: Short-Form Health Survey; PCS: Physical Component Score; MCS: Mental Component Score; PASE: Physical Activity Scale for the Elderly; DQOL: Diabetes Quality Of Life; NCR: Normal Clinical Range; NT-proBNP: N-Terminal Prohormone of Brain Natriuretic Peptide; LDL: Low-Density Lipoprotein; HDL: High-Density Lipoprotein.

**Table 2:** Comparison of characteristics between PA improvers and non-improvers considering the MCID of 600 steps/day.

Variables	Improved (n = 24)	Not Improved (n = 34)	*p*	Effect Size (95%CI)
**Sex**				
Male	20 (83%)	29 (85%)	0.84	
Female	4 (17%)	5 (15%)		
Age, years	68.75 ± 8.88	73.12 ± 6.73	0.04	−0.57 (−1.10; −0.03)
BMI, kg/$m^{2}$	27.39 ± 5.58	28.44 ± 4.00	0.17	
Number of drugs				
≤2	7 (29%)	8 (24%)	0.82	
3	6 (25%)	11 (32%)		
4	6 (25%)	10 (29%)		
≥5	5 (21%)	5 (15%)		
NYHA classification				
1	1	0	0.33	
2	15	20		
3	7	14		
4	1	0		
EF, %	48.44 ± 12.18	48.78 ± 9.59	0.94	
Daily step count, n	6295 (3269–9563)	7982 (3368–10,357)	0.75	
6MWT Meters % of predicted value	444.13 ± 99.29 90.77 ± 18.80	399.47 ± 119.55 86.00 ± 23.65	0.20 0.68	
**Questionnaires**, score				
MLHFQ	8 (2.516)	10.5 (223)	0.42	
SF-12: PCS	46.66 ± 8.12	39.74 ± 10.33	0.01	0.73 (0.19;1.27)
SF12: MCS	55.82 ± 5.33	54.08 ± 7.19	0.32	
PASE	78.92 (52.44–121.8)	79.96 (54.29–118.14)	0.76	
DQOL Section 1	26.88 ± 7.40	29.12 ± 8.10	0.29	
DQOL Section 2	27 (2430)	26.5 (2530)	0.89	
DQOL Section 3	6 (57.5)	6 (58)	0.80	
**Blood chemistry test**				
Glycemia, mg/dL	122(108.5–138)	138 (129–150)	0.10	
Glycated hemoglobin, mmol/mol	47.5 (44–50)	54.5 (48–64)	0.02	0.23 (−0.03;0.44)
NT-proBNP, pg/mL	938.4 (173.5–1015)	771.5 (178–1001)	0.91	
Renal clearance, mL/min	69.87 ± 19.45	52.04 ± 23.31	<0.01	0.82 (0.27;1.36)
Creatinine, mg/dL	1.2 (0.96–1.44)	1.3 (1.09–1.47)	0.20	
Total cholesterol, mg/dL	120 (113–146)	125 (105–152)	0.68	
LDL cholesterol, mg/dL	57.5 (52.25–73.5)	59.5 (46–85)	0.79	
HDL cholesterol, mg/dL	43 (40.5–53.85)	41.5 (36–45)	0.06	

Legend: Data are presented as mean ± SD or median (1st–3rd quartile) or number (%). BMI: Body Mass Index; CI: Comorbidity Index; NYHA: New York Heart Association Functional Classification; EF: Ejection Fraction; 6MWT: 6-Minute Walking Test; MLHFQ: Minnesota Living With Heart Failure Questionnaire; SF-12: Short-Form Health Survey; PCS: Physical Component Score; MCS: Mental Component Score; PASE: Physical Activity Scale for the Elderly; DQOL: Diabetes Quality Of Life; NT-proBNP: N-Terminal Prohormone of Brain Natriuretic Peptide. Improved: patients who have increased their physical activity by at least 600 steps per day on average. Not improved: patients who have not improved their physical activity. Effect size was calculated only in cases with a significant *p*-value according to Cohen’s d for the mean and the rank biserial correlation for median values.

**Table 3:** Multivariate linear regression model explaining PA (above) and PF (below) following the TMP intervention.

**Y = Physical Activity (Daily step count**) R^2^ = 0.6580 F (4.53) = 25.49 *p* < 0.001					
	**Variable**	**B**	**95% CI**	**β**	***p***
	Intercept	4489.77	−6282.7; 15,262.2		0.41
	6MWT_T0	8.5	−0.3; 17.23	0.1931	0.06
	Age	−144.0	−262.1; −25.90	−0.2305	0.02
	Daily step count_T0	0.5967	0.3734; 0.8199	0.5004	<0.001
	HDL_cholesterol	119.7	39.07; 200.31	0.2426	<0.001
**Y = Physical Fitness (Meters at 6MWT)** R^2^ = 0.9007 F (3.54) = 163.29 *p* < 0.001					
	**Variable**	**B**	**95% CI**	**β**	***p***
	Intercept	325.1	161.2; 489.1		<0.001
	BMI	−2.04	−4.30; 0.23	−0.079	0.08
	Age	−2.86	−4.40; 1.32	−0.1885	<0.001
	6MWT_T0	0.90	0.79; 1.00	0.8431	<0.001

Legend: 6MWT_T0; 6-Minute Walking Test at admission; BMI: body mass index; HDL: high-density lipoprotein.

## Data Availability

The raw data supporting the conclusions of this article will be made available by the authors upon request.

## Abbreviations

The following abbreviations are used in this manuscript:

6MWT	6-Minute Walking Test
BMI	Body Mass Index
Bw	B Weighted
CI	Comorbidity Index
CIRS	Cumulative Illness Rating Scale
DQOL	Diabetes Quality Of Life
EF	Ejection Fraction
HDL	High-Density Lipoprotein
HP	Healthcare Professional
HF	Heart Failure
LDL	Low-Density Lipoprotein
MCID	Minimal Clinically Important Difference
MCS	Mental Component Score of SF12
MLHFQ	Minnesota Living With Heart Failure Questionnaire
NT-proBNP	N-Terminal Prohormone of Brain Natriuretic Peptide
NYHA	New York Heart Association Functional Classification
PA	Physical Activity
PASE	Physical Activity Scale for the Elderly
PCS	Physical Component Score of SF12
PF	Physical Fitness
RCT	Randomized Controlled Trial
SF-12	Short-Form Health Survey
SI	Severity Index
TMPs	Telemedicine Programs
T2DM	Type 2 Diabetes Mellitus

## References

[1] McDonagh T.A., Metra M., Adamo M., Gardner R.S., Baumbach A., Böhm M., Burri H., Butler J., Čelutkienė J., Chioncel O. (2021). ESC Scientific Document Group (2021). 2021 ESC Guidelines for the diagnosis and treatment of acute and chronic heart failure. Eur. Heart J..

[2] Erqou S., Lee C.T., Suffoletto M., Echouffo-Tcheugui J.B., de Boer R.A., van Melle J.P., Adler A.I. (2013). Association between glycated haemoglobin and the risk of congestive heart failure in diabetes mellitus: Systematic review and meta-analysis. Eur. J. Heart Fail..

[3] Seferović P.M., Petrie M.C., Filippatos G.S., Anker S.D., Rosano G., Bauersachs J., Paulus W.J., Komajda M., Cosentino F., de Boer R.A. (2018). Type 2 diabetes mellitus and heart failure: A position statement from the Heart Failure Association of the European Society of Cardiology. Eur. J. Heart Fail..

[4] Thrainsdottir I.S., Aspelund T., Thorgeirsson G., Gudnason V., Hardarson T., Malmberg K., Sigurdsson G., Rydén L. (2005). The association between glucose abnormalities and heart failure in the population-based Reykjavik study. Diabetes Care.

[5] Cavender M.A., Steg P.G., Smith S.C., Eagle K., Ohman E.M., Goto S., Kuder J., Im K., Wilson P.W., REACH Registry Investigators (2015). Impact of Diabetes Mellitus on Hospitalization for Heart Failure, Cardiovascular Events, and Death: Outcomes at 4 Years from the Reduction of Atherothrombosis for Continued Health (REACH) Registry. Circulation.

[6] Hordern M.D., Coombes J.S., Cooney L.M., Jeffriess L., Prins J.B., Marwick T.H. (2009). Effects of exercise intervention on myocardial function in type 2 diabetes. Heart.

[7] Kanaley J.A., Colberg S.R., Corcoran M.H., Malin S.K., Rodriguez N.R., Crespo C.J., Kirwan J.P., Zierath J.R. (2022). Exercise/Physical Activity in Individuals with Type 2 Diabetes: A Consensus Statement from the American College of Sports Medicine. Med. Sci. Sports Exerc..

[8] Messina G., Alioto A., Parisi M.C., Mingrino O., Di Corrado D., Crescimanno C., Kuliś S., Nese Sahin F., Padua E., Canzone A. (2023). Experimental study on physical exercise in diabetes: Pathophysiology and therapeutic effects. Eur. J. Transl. Myol..

[9] Dugal J.K., Malhi A.S., Ramazani N., Yee B., DiCaro M.V., Lei K. (2024). Non-Pharmacological Therapy in Heart Failure and Management of Heart Failure in Special Populations-A Review. J. Clin. Med..

[10] Izawa K.P., Watanabe S., Oka K., Hiraki K., Morio Y., Kasahara Y., Brubaker P.H., Osada N., Omiya K., Shimizu H. (2013). Usefulness of step counts to predict mortality in Japanese patients with heart failure. Am. J. Cardiol..

[11] Izawa K.P., Watanabe S., Oka K., Osada N., Omiya K., Brubaker P.H., Shimizu H. (2013). Diabetes mellitus may lower daily physical activity in heart failure patients. Int. J. Cardiol..

[12] Vetrovsky T., Siranec M., Frybova T., Gant I., Svobodova I., Linhart A., Parenica J., Miklikova M., Sujakova L., WATCHFUL Investigators (2024). Lifestyle Walking Intervention for Patients with Heart Failure with Reduced Ejection Fraction: The WATCHFUL Trial. Circulation.

[13] Freene N., McManus M., Mair T., Tan R., Davey R. (2020). High sedentary behaviour and low physical activity levels at 12 months after cardiac rehabilitation: A prospective cohort study. Ann. Phys. Rehabil. Med..

[14] Dibben G.O., Dalal H.M., Taylor R.S., Doherty P., Tang L.H., Hillsdon M. (2018). Cardiac rehabilitation and physical activity: Systematic review and meta-analysis. Heart.

[15] Leale I., Figlioli F., Giustino V., Brusa J., Barcellona M., Nocera V., Canzone A., Patti A., Messina G., Barbagallo M. (2024). Telecoaching as a new training method for elderly people: A systematic review. Aging Clin. Exp. Res..

[16] Loeckx M., Rabinovich R.A., Demeyer H., Louvaris Z., Tanner R., Rubio N., Frei A., De Jong C., Gimeno-Santos E., Rodrigues F.M. (2018). Smartphone-Based Physical Activity Telecoaching in Chronic Obstructive Pulmonary Disease: Mixed-Methods Study on Patient Experiences and Lessons for Implementation. JMIR Mhealth Uhealth.

[17] Bernocchi P., Giudici V., Borghi G., Bertolaia P., D’Isa S., Trevisan R., Scalvini S. (2024). Telemedicine home-based management in patients with chronic heart failure and diabetes type II: Study protocol for a randomized controlled trial. Trials.

[18] Vitacca M., Paneroni M. (2025). Are Oxygen and Non-Invasive Ventilation Useful “Clean Doping Boosters” for Thoracoabdominal Asynchrony During Exertion in Severe COPD?. Arch. Bronconeumologia.

[19] Holland A.E., Spruit M.A., Troosters T., Puhan M.A., Pepin V., Saey D., McCormack M.C., Carlin B.W., Sciurba F.C., Pitta F. (2014). An official European Respiratory Society/American Thoracic Society technical standard: Field walking tests in chronic respiratory disease. Eur. Respir. J..

[20] Enright P.L., Sherrill D.L. (1998). Reference equations for the six-minute walking in healthy adults. Am. J. Respir. Crit. Care Med..

[21] Mantovani A.M., Duncan S., Codogno J.S., Lima M.C., Fernandes R.A. (2016). Different Amounts of Physical Activity Measured by Pedometer and the Associations with Health Outcomes in Adults. J. Phys. Act. Health.

[22] Tang C.Y., Bernstein B., Blackstock F., Blondeel A., Gershon A., Gimeno-Santos E., Gloeckl R., Marques A., Spruit M.A., Garvey C. (2024). Unravelling the complex interplay of factors behind exercise limitations and physical inactivity in COPD. Breathe.

[23] Demeyer H., Burtin C., Hornikx M., Camillo C.A., Van Remoortel H., Langer D., Janssens W., Troosters T. (2016). The Minimal Important Difference in Physical Activity in Patients with COPD. PLoS ONE.

[24] Shingai K., Matsuda T., Kondoh Y., Kimura T., Kataoka K., Yokoyama T., Yamano Y., Ogawa T., Watanabe F., Hirasawa J. (2023). Physical activity in idiopathic pulmonary fibrosis: Longitudinal change and minimal clinically important difference. Chron. Respir. Dis..

[25] Shoemaker M.J., Curtis A.B., Vangsnes E., Dickinson M.G. (2013). Clinically meaningful change estimates for the six-minute walking test and daily activity in individuals with chronic heart failure. Cardiopulm. Phys. Ther. J..

[26] Dunbar S.B., Reilly C.M., Gary R., Higgins M.K., Culler S., Butts B., Butler J. (2015). Randomized clinical trial of an integrated self-care intervention for persons with heart failure and diabetes: Quality of life and physical functioning outcomes. J. Card. Fail..

[27] Heimer M., Schmitz S., Teschler M., Schäfer H., Douma E.R., Habibovic M., Kop W.J., Meyer T., Mooren F.C., Schmitz B. (2023). Ehealth for maintenance cardiovascular rehabilitation: A systematic review and meta-analysis. Eur. J. Prev. Cardiol..

[28] Skivington K., Matthews L., Simpson S.A., Craig P., Baird J., Blazeby J.M., Boyd K.A., Craig N., French D.P., McIntosh E. (2021). A new framework for developing and evaluating complex interventions: Update of Medical Research Council guidance. Br. Med. J..

[29] Han X., Li P., Yang Y., Liu X., Xia J., Wu W. (2021). An Exploration of the Application of Step Counter-Based Physical Activity Promotion Programs in Patients with Chronic Obstructive Pulmonary Disease: A Systematic Review. Front. Public Health.

[30] Jake-Schoffman D.E., White T.I., Lavoie H.A., Monroe C.M., Christou D.D. (2024). Cadence as a Behavioral Target in Physical Activity Interventions: A Narrative Review. Am. J. Lifestyle Med..

[31] Kasiak P.S., Wiecha S., Cieśliński I., Takken T., Lach J., Lewandowski M., Barylski M., Mamcarz A., Śliż D. (2023). Validity of the Maximal Heart Rate Prediction Models among Runners and Cyclists. J. Clin. Med..

